# Correction: Stem cell transcriptional profiles from mouse subspecies reveal *cis*-regulatory evolution at translation genes

**DOI:** 10.1038/s41437-024-00730-0

**Published:** 2024-10-24

**Authors:** Noah M. Simon, Yujin Kim, Joost Gribnau, Diana M. Bautista, James R. Dutton, Rachel B. Brem

**Affiliations:** 1 Biology of Aging Doctoral Program, Leonard Davis School of Gerontology, University of Southern California, Los Angeles, CA 90089 USA; 2 Buck Institute for Research on Aging, Novato, CA 94945 USA; 3 Department of Plant and Microbial Biology, University of California, Berkeley, Berkeley, CA 94720 USA; 4 Stem Cell Institute, University of Minnesota, Minneapolis, MN 55455 USA; 5 Department of Genetics, Cell Biology, and Development, University of Minnesota, Minneapolis, MN 55455 USA; 6 Department of Reproduction and Development, Erasmus MC, Rotterdam, PO Box 2040, CA 3000 Netherlands; 7 Department of Molecular and Cell Biology, University of California, Berkeley, Berkeley, CA 94720 USA

**Keywords:** Evolutionary genetics, Gene regulation

Correction to: *Heredity* 10.1038/s41437-024-00715-z, published online 20 August 2024

In this article Joost Gribnau at affiliation Department of Reproduction and Development, Erasmus MC, PO Box 2040, 3000 CA Rotterdam, Netherlands was added to the author list.

The original article has been corrected.

