# Supplementary material for: Stem cell transcriptional profiles from mouse subspecies reveal cis-regulatory evolution at translation genes
Source: Heredity (Edinb). 2024 Aug 20;133(5):308–16. doi: 10.1038/s41437-024-00715-z (PMC11527988; doi:10.1038/s41437-024-00715-z)
Supplement: Supplementary file 1 — Supplementary information [file 41437_2024_715_MOESM1_ESM.docx]

# Supplementary figure captions

**Figure S1. Directional *cis*-regulatory variation in translation gene expression in stem cells between mouse subspecies.** Data are as in Figure 1B **(A-B)** or Figure 1C **(C-I)** of the main text except that transcriptomes analyzed were as follows. **(A)** CAST x 129 hybrid stem cells (Marks et al. 2015). **(B)** CAST x BL6 hybrid stem cells (Werner et al. 2017). **(C)** CAST and 129 homozygous stem cells (Skelly et al. 2020). **(D)** CAST and BL6 homozygous stem cells (Skelly et al. 2020). **(E)** CAST and WSB/EiJ homozygous stem cells (Skelly et al. 2020). **(F)** CAST and PWD/PhJ homozygous stem cells (Skelly et al. 2020). **(G)** CAST and A/J homozygous stem cells (Skelly et al. 2020). **(H)** CAST and NOD/ShiLtJ homozygous stem cells (Skelly et al. 2020). **(I)** CAST and NZO/HILtJ homozygous stem cells (Skelly et al. 2020).

**Figure S2. Ehmt2 knockout induces translation genes.** Data are as in Figure 3C of the main text except that transcriptomes analyzed were wild-type and Ehmt2 knockout stem cells (Auclair et al. 2016).

# Supplementary table captions

**Table S1. Allele-specific normalized read counts from RNA-seq of stem cells from hybrids involving CAST.** Each tab reports allele-specific expression measurements in one genotype of hybrid embryonic stem cells. Tab TPM_GSE60738_CAST_M_x_129_F contains results from four replicate transcriptomes of replicates of CAST/EiJ male x 129/SvImJ female F1 hybrid cells from (Marks et al. 2015). Tab TPM_GSE234761_CAST_M_x_129_F contains results from four replicate transcriptomes of 129Cas (CAST/EiJ male x 129/SvImJ female F1 hybrid) cells from this study. Tab TPM_GSE90516_CAST_M_x_BL6_F contains results from 12 replicate transcriptomes of CAST/EiJ male x C57BL/6J female cells (Werner et al. 2017). Tab TPM_GSE90516_CAST_F_x_BL6_M contains results from 11 replicate transcriptomes of CAST/EiJ female x C57BL/6J male cells (Werner et al. 2017). In each tab, the first column reports Ensembl gene accession numbers. The remaining columns are TPM (transcripts per million) values for reads mapped non-ambiguously to either parental allele for each gene (two columns for each biological sample).

**Table S2. Normalized read counts from RNA-seq of stem cells from homozygous strains.** Each tab reports expression measurements from one data source of experimental profiles of cell culture from homozygous mouse strains: pluripotent stem cells (PSCs) of 129 and CAST/EiJ genotypes from this study; embryonic stem cells of eight genotypes from (Skelly et al. 2020); *Ctr9* knockdown in embryonic stem cells (Ruan et al. 2023); and *Ehmt2* knockouts in E8.5 embryos (Auclair et al. 2016). In each tab, the first column reports Ensembl gene accession numbers. The remaining columns report the TPM (transcripts per million) values. Tab TPM_GSE234761_homozyg_CAST_129 contains results from two replicate transcriptomes of CAST PSCs and four replicate transcriptomes of 129 PSCs. Tab TPM_E_MTAB_7730_8_strains contains results from ESC replicate transcriptomes from eight strains (3 replicates CAST, 35 replicates PWD, 3 replicates WSB, 36 replicates BL6, 3 replicates 129, 3 replicates AJ, 22 replicates NOD, 3 replicates NZO). Tab TPM_GSE219206_CTR9_KD contains results from two replicate transcriptomes of control shRNA ESC samples and two replicate transcriptomes of shRNA knockdown of *Ctr9,* Tab TPM_GSE71500_EHMT2_KO contains results from two replicate transcriptomes of wild-type embryos (E8.5) and two replicate transcriptomes of *Ehmt2* knockout embryos (E8.5).

**Table S3. Mapping results from simulated RNA-seq experiments using hybrid genomes.** The first and second tabs report the mapped read counts from simulated RNA-seq of CAST x 129 and CAST x BL6 hybrids, respectively. In a given tab, for each row, the first column lists the Ensembl gene id for the gene analyzed; the second through fourth columns report the mapping percentage (the number of simulated reads mapped, normalized by the total number of simulated reads) for the indicated allele in the indicated simulated replicate; the last column reports the absolute value of the difference of mapping percentages between the CAST allele and that of the indicated strain (129 or BL6).

**Table S4. Accession ID numbers for gene homologs across strains.** Gene and transcript accession numbers used to compare homologous alleles between strains. GRCm38_ensembl_gene_ID, Ensembl gene IDs for the C57BL/6 reference. 129_MGP_gene_ID, 129S1/SvImJ Mouse Genome Project IDs. CAST_MGP_gene_ID, CAST/EiJ Mouse Genome Project IDs. gene_name, gene names from the Mouse Genome Informatics database. GRCm38_ensembl_transcript_ID is the Ensembl transcript ID for the longest in-frame ORF for that gene; empty cells in this column are genes with no valid ORF in the GRCm38 genome using the Ensembl build 102 annotations.

**Table S5. Differential expression test results in hybrid and homozygous stem cells.** Each tab reports results of a genome-wide survey of differential expression between homologous alleles in interspecies hybrid stem cells; between homozygote stem cells of different species genotypes; or between a regulator gene knockout and its isogenic wild-type in stem cell or early embryo samples. Log2FoldChange, log-transformed ratio of normalized expression of the indicated alleles or homozygote samples; Pvalue, Wald test *p*-value; Padj, Benjamini-Hochberg adjusted *p*-value. Tab DESeq_GSE60738_CASTx129_hyb, comparison of CAST vs 129 allele-specific expression in F1 hybrid stem cells from (Marks et al. 2015). Tab DESeq_GSE234761_CASTx129_hyb, comparison of CAST vs 129 allele-specific expression in F1 hybrid stem cells from this study. Tab DESeq_GSE90516_CAST_x_BL6_hyb, comparison of CAST vs BL6 allele-specific expression in F1 hybrid stem cells from (Werner et al. 2017). Tab DESeq_GSE234761_CASTvs129, comparison of CAST vs 129 expression in homozygous stem cells from this study. Tab DESeq_E_MTAB_7730_CASTvs129, comparison of CAST vs 129 expression in homozygous stem cells from (Skelly et al. 2020). Tab DESeq_E_MTAB_7730_CASTvsBL6, comparison of CAST vs BL6 expression in homozygous stem cells from (Skelly et al. 2020). Tab DESeq_E_MTAB_7730_CASTvsWSB, comparison of CAST vs WSB expression in homozygous stem cells from (Skelly et al. 2020). Tab DESeq_E_MTAB_7730_CASTvsPWD, comparison of CAST vs PWD expression in homozygous stem cells from (Skelly et al. 2020). Tab DESeq_E_MTAB_7730_CASTvsAJ, comparison of CAST vs AJ expression in homozygous stem cells from (Skelly et al. 2020). Tab DESeq_E_MTAB_7730_CASTvsNOD, comparison of CAST vs NOD expression in homozygous stem cells from (Skelly et al. 2020). Tab DESeq_E_MTAB_7730_CASTvsNZO, comparison of CAST vs NZO expression in homozygous stem cells from (Skelly et al. 2020). Tab DESeq_GSE219206_Ctr9_KD, comparison of control shRNA vs *Ctr9* knockdown expression in stem cells from (Ruan et al. 2023). Tab DESeq_GSE71500_EHMT2_KO, comparison of wildtype vs *Ehmt2* knockout expression in E8.5 embryos from (Auclair et al. 2016).

**Table S6. A screen for directional allele-specific expression in CAST x 129 hybrid embryonic stem cells.** Each row reports results of a statistical test for directional allele-specific expression variation in CAST/EiJ male x 129/SvImJ female F1 hybrid embryonic stem cells (Marks et al. 2015) in the indicated Gene Ontology term. total_number_of_genes, number of genes in the term with analyzable expression measurements; sum_sign_statistic, sum across genes of the term of log_2_(129 allele expression/CAST allele expression); resampling_P_val, resampling-based significance of the enrichment for high absolute value of the sign statistic in the term; Adjusted_P, *p*-value after Benjamini-Hochberg correction for multiple testing.

**Table S7. Patterns of variation between mouse subspecies in binding sites of transcriptional regulators upstream of translation genes.** Data are as in Table 1 of the main text, except that results from all tested regulators are shown.

**Table S8. Counts of divergent and polymorphic sites in binding sites of top four candidate transcriptional regulators.** Each row reports counts upstream of a gene, of sequence variation between wild mouse populations in a binding site for Ctr9, Rfx6, Hoxa11, or Ehmt2 from the GTRD database (Kolmykov et al. 2021). The first column reports the Ensembl transcript ID. The next three columns report the chromosome, strand and transcription start site (TSS) position for that gene, respectively. Column E reports whether or not the gene is part of the translation GO term. Column F reports the transcription factor to the binding site for that row. Columns G-H report the chromosomal positions of the start and end of the binding site, respectively. Columns I-J report the upstream distance of the binding site start and end, respectively, relative to the TSS. Column K reports the length of the binding site which fall within the first 50kb upstream of the TSS. The remaining pairs of columns report the divergent sites within the binding site, d_TF_, or the number of polymorphic sites within the binding site, p_TF_**,** from a comparison between a *M. m. castaneus* population (captured in northwest India) and one population of another subspecies: *M. m. domesticus* populations from France (Mmd_FRA), Germany (Mmd_GER), and Iran (Mmd_IRA); or *M. m. musculus* populations from Kazakhstan (Mmm_KAZ), the Czech Republic (Mmm_CZE), and Afghanistan (Mmm_AFG).
