# Supplementary figures and images for: Stem cell transcriptional profiles from mouse subspecies reveal cis-regulatory evolution at translation genes
Source: Heredity (Edinb). 2024 Aug 20;133(5):308–16. doi: 10.1038/s41437-024-00715-z (PMC11527988; doi:10.1038/s41437-024-00715-z)

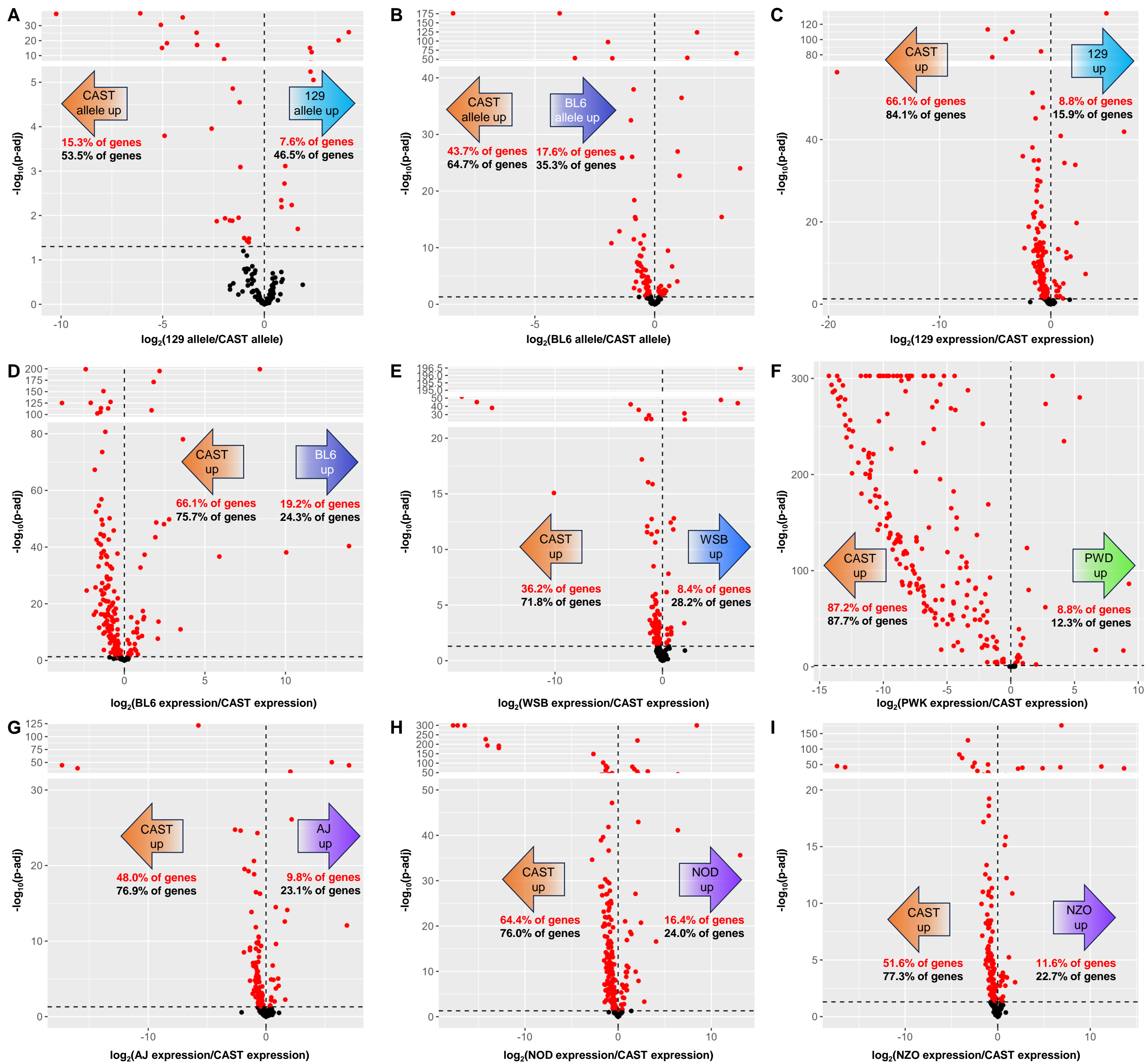

Supplement: Supplementary file 2 — Figure S1 [file 41437_2024_715_MOESM2_ESM.pdf]

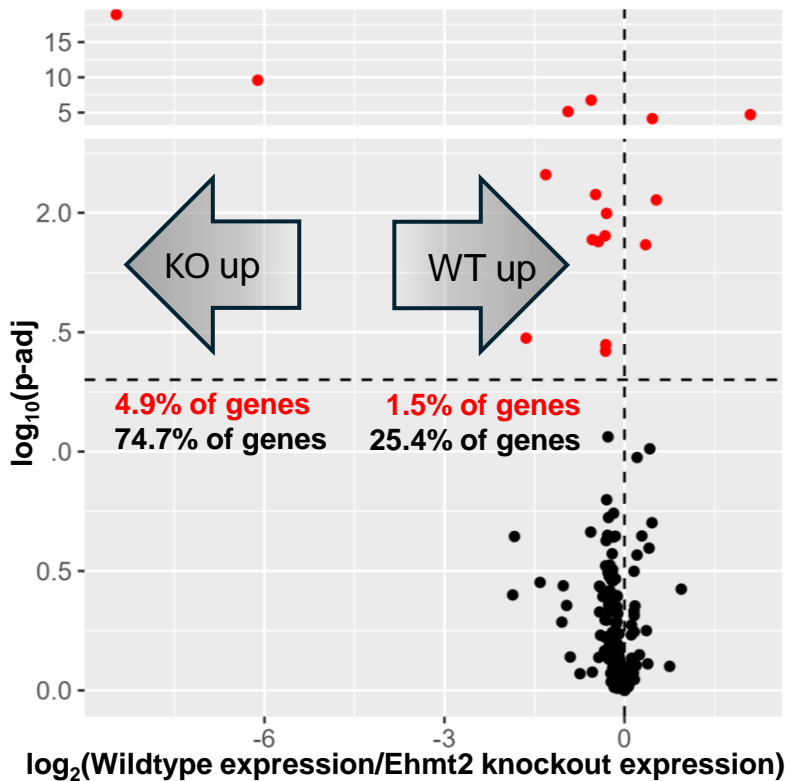

Supplement: Supplementary file 3 — Figure S2 [file 41437_2024_715_MOESM3_ESM.pdf]
